# Barriers to, and facilitators of, parenting programmes for childhood behaviour problems: a qualitative synthesis of studies of parents’ and professionals’ perceptions

**DOI:** 10.1007/s00787-013-0401-2

**Published:** 2013-04-06

**Authors:** J. Koerting, E. Smith, M. M. Knowles, S. Latter, H. Elsey, D. C. McCann, M. Thompson, E. J. Sonuga-Barke

**Affiliations:** 1 Developmental Brain-Behaviour Laboratory, Institute for Disorders of Impulse and Attention, School of Psychology, University of Southampton, Highfield Campus, Southampton, SO17 1BJ UK; 2 OCD Research Group, Faculty of Health Sciences, University of Southampton, Southampton, UK; 3 Academic Unit of Public Health, University of Leeds, Leeds, UK; 4 Department of Experimental Clinical and Health Psychology, University of Ghent, Ghent, Belgium

**Keywords:** Qualitative methods, Parent training, ADHD, Conduct disorder, Oppositional defiant disorder, Behaviour problems, Treatment barriers, Hard-to-reach

## Abstract

Disruptive behaviour problems (DBPs) during childhood exert a high burden on individuals, families and the community as a whole. Reducing this impact is a major public health priority. Early parenting interventions are recommended as valuable ways to target DBPs; however, low take-up of, and high drop-out rates from, these programmes seriously reduce their effectiveness. We present a review of published qualitative evidence relating to factors that block or facilitate access and engagement of parents with such programmes using a thematic synthesis approach. 12 papers presenting views of both parents and professionals met our inclusion and quality criteria. A large number of barriers were identified highlighting the array of challenges parents can face when considering accessing and engaging with treatment for their child with behavioural problems. Facilitating factors in this area were also identified. A series of recommendations were made with regard to raising awareness of programmes and recruiting parents, providing flexible and individually tailored support, delivering programmes through highly skilled, trained and knowledgeable therapists, and highlighting factors to consider when delivering group-based programmes. Clinical guidelines should address barriers and facilitators of engagement as well as basic efficacy of treatment approaches.

**Electronic supplementary material:**

The online version of this article (doi:10.1007/s00787-013-0401-2) contains supplementary material, which is available to authorized users.

## Background

Disruptive behaviour problems (DBPs), including Attention-Deficit/Hyperactivity Disorder (ADHD), Conduct Disorder (CD), and Oppositional Defiant Disorder (ODD) represent a major long-term burden to children, families, and the community at large. They are prevalent in community samples all around the developed world [1–3]; are common reasons for referral to youth mental health clinics [2, 4]; are associated with significant impairment and maladjustment [5]; and have become a considerable source of public health concern [6, 7]. Long-term outcomes include academic underachievement and underemployment, juvenile delinquency, adult crime and violence, anti-social behaviour problems, and substance misuse [8, 9]. They significantly impinge on public sector costs—by age 28 individuals with CD have, on average, cost the state ten times more than those without [10]; are disproportionally represented in the Criminal Justice System [11]; and have higher costs in educational, medical, and mental health sectors, outpatient mental health clinics and health-care providers [12–14].

Treatment of DBPs often begins during the school years once the condition is well established [15]. Medication and psycho-social interventions are available [16, 17] although where children do not have ADHD, medication is rarely used and treatment approaches rely more on psychological approaches [18]. Behavioural-psychosocial treatments, on their own, are regarded as the most appropriate front-line treatment with younger children even when ADHD is present; except in exceptional circumstances [19]. Non-pharmacological treatments include behaviour therapy, parent training (PT), and cognitive therapy [3]. Parenting components are considered to be important in all child-centred treatment choices, where parents reinforce appropriate child behaviours and promote positive interactions [3]. A wide variety of PT programmes are available and evidence from systematic reviews [20, 21] shows that they improve a range of outcomes including parent and child well-being, parent–child interactions, decreased maternal depression and stress and child non-compliance and aggression. However, effects with regard to ADHD specifically are less well established [22].

Behavioural approaches may be especially effective if implemented in preschool through PT programmes [23]. If left untreated DBPs become less responsive to intervention [24, 25]. However, while early behavioural interventions are efficacious in randomised controlled trials, their effectiveness in the real world is limited by a number of factors that affect take up and continued engagement with PT programmes. For example, 30–68 % of families with children who have DBPs have been found to decline to take part in available programmes [26, 27]; and out of 60 % of families interested in PT programmes in the UK, only 4–18 % are estimated to have taken them up [21]. Even where families take up the offer of a programme, dropout rates are estimated at up to 40 % for PT programmes [28, 29] and 40–60 % for child mental health services more generally [30, 31]. When parents receive monetary compensation for attending, average completion rates are still below 60 % [32]. The situation is worse when families are “difficult to engage” or “hard to reach”. These families generally fall under three categories: “minority groups”, those “slipping through the net” and the “service resistant” [33]. Membership of “hard to reach” populations is predicted by child, parental, cultural, and socioeconomic factors (see [34] for a review).

Effective planning and targeting of services requires information about parents’ and stakeholders’ views concerning the reasons for low uptake and completion of parenting programmes. Qualitative approaches have been used to gather such information and are considered most appropriate for generating valuable information to inform clinical decision-making and policy development [35]. Systematic review of qualitative research provides an important technology for developing future policy and practice to bring research closer to decision making [36, 37]. The value of synthesising qualitative research in order to facilitate appropriate and effective healthcare is being increasingly recognised [38]. To date, the authors know of no meta-synthetic qualitative review that has examined these issues. The objective of the current research was therefore to systematically review and synthesise qualitative studies regarding the perceptions about barriers and facilitators to PT programmes of those centrally engaged with their delivery. The analysis was focused on both access and continued engagement with PT programmes used for the treatment of DBPs in children. Views of both parents and professionals were included.

## Methods

A systematic literature search was conducted in 12 databases (ISI Web of knowledge, EMBASE, Cinahl, JSTOR, Social Services Abstracts, Wiley, ERIC, Science Direct, Psych Articles, Psych Info, Medline, Cochrane Reviews). The search terms used for Medline are detailed in Appendix I (available online). These search terms were adapted for all other databases. Systematic literature searches often do not yield comprehensive results for qualitative studies [39, 40], therefore, additional Google/Google Scholar searches were performed and relevant government websites were interrogated. Furthermore, included articles were searched for relevant references and citations, and a number of journals were hand searched.

### Inclusion criteria and quality assessment

**Fig. 1:**
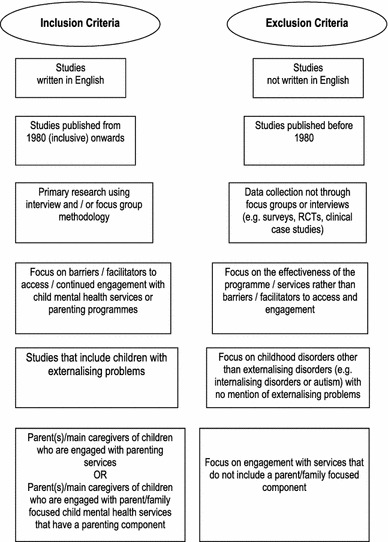
Inclusion and exclusion criteria for quality assessment

**Fig. 2:**
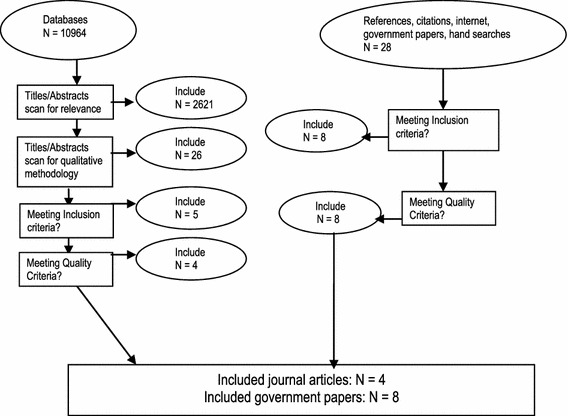
Literature selection process

Initial searches produced 10,992 papers (Figs. 1, 2). All titles were initially scanned for relevance by two of the authors (JK and ES) separately. At this stage, all studies that were about treatment for children with behavioural problems were included (*N* = 2,621). Interrater reliability was calculated on a sample of 100 papers and an excellent Intra-class Correlation (0.89) was established. The full inclusion criteria were then applied to the abstracts by JK and ES (Intra-class Correlation = 0.91). After this stage, the full texts were obtained and all further decisions regarding inclusion criteria were made by JK and ES together.

Papers were included if they both met all inclusion criteria (Fig. 1) and EPPI-Centre quality criteria [41, 42]: These were that; (1) the research question was clearly stated; (2) the method of analysis was appropriate; (3) steps were taken to increase rigour in the sampling; (4) steps were taken to increase rigour in the data collected (e.g. through use of semi-structured interview schedules to ensure reliability and use of pilot interviews to increase validity); (5) steps were taken to increase rigour in the analysis of the data (e.g. through using independent coders to increase reliability and search for negative cases to ensure validity); (6) the findings of the study were grounded in and supported by the data; (7) the findings of the study had sufficient breadth; and (8) the findings of the study had sufficient depth. The quality criteria were first applied independently by two of the authors (JK, ES) to each of the studies. Disagreements were solved first through discussions between JK and ES. Where there was any uncertainty another member of the team (DCM) was consulted, which was the case for three papers. One paper was excluded due to poor quality. All papers were classified as “robust” (if they fulfilled at least five of the above criteria) or “less robust”.

### Characteristics of included studies

Twelve papers were included in the synthesis. The characteristics of included studies are provided in Table 1. Six studies were conducted in the UK, three in Australia, two in the US and one in Canada. Eight studies collected data using individual interviews, two used focus groups and two used both interview and focus group data. Four studies collected data from both parents and professionals, five studies used data from parents alone and three used data from professionals only. There were 353 participants in total—171 parents/caregivers^1^ of children aged 2–17 years, and 202 professionals involved in the delivery of PT programmes, health services, social work services and/or working with ‘hard to reach’ families (see Table 1 for participant details). Four of the nine “parent” studies were conducted with ‘hard to reach’ groups [fathers, parents living in rural areas and from culturally and linguistically diverse (CALD) backgrounds] and four used data from parents who had dropped out of programmes early or had not attended. Six studies included views about group-based behavioural PT programmes. All the other studies included views about a wider range of parent-based interventions and did not explicitly state whether the interventions were group or individual-based.

**Table 1:** Profile of studies

Study ID, reference, study location	Data collection and analysis methods	Participant characteristics	Focus/topic	Results	Reliability and validity of data collection methods	Reliability and validity of data analysis methods	Robustness
I Law et al. [52] UK	6 focus groups run separately for parents and professionals Content analysis	17 parents using parenting services 24 professionals representing education, health and social work services	Barriers to service access Parenting interventions targeting infant mental health, emotional and behavioural difficulties and ADHD	*Barriers identified by parents*: Situational barriers (location) Needs not recognised by professionals Therapist qualities and background *Barriers identified by professionals* Programme content Inter-agency collaboration *Barriers identified by both parents & professionals* Delay accessing information/support Information not accessible for parents with additional needs	*Reliability*: Interviews recorded and transcribed, field notes taken. *Validity*: Not stated	*Reliability*: Use of transcribed data *Validity*: Not stated	Robust
II Boydell et al. [53] Canada	Individual interviews Not stated	30 parents of children (aged 3–17 yrs) formally diagnosed with emotional or behavioural disorders.	Barriers/facilitators to service access Hard to reach groups (rural) Access issues associated with mental health care for children and youth in rural communities	*Personal barriers* (stigma, lack of information, financial difficulties) *Personal facilitators* (word of mouth & advocacy) *Systemic barriers* (human resources, policy & funding issues, waiting time, invisibility) *Systemic facilitators* (personalised services, offering services within local communities) *Environmental barriers* (distance) *Environmental facilitators* (small size community)	*Reliability*: Interview schedule Interviews recorded and transcribed *Validity*: Interview schedule reviewed by advisory committee consisting of academics, service providers and health policy makers	*Reliability*: Use of transcribed data *Validity*: Several researchers examined transcripts. Coding structure was developed through team discussions.	Robust
III Bayley [54] UK	2 focus groups with fathers 9 individual interviews with professionals Thematic analysis	14 fathers engaged in services 9 professionals & academic experts in parenting programmes or working with fathers	Barriers/facilitators to service access Hard to reach groups (fathers)	*Barriers to service access* Lack of information Situational barriers Mother-orientated environment Additional needs *Facilitators to service access* Advertising, flexible & alternative forms of provision, relationship with therapist, father-focused organisational approaches, programme content, support for additional needs	*Reliability* Not stated *Validity* Not stated	*Reliability* Not stated *Validity* Not stated	Less robust
IV Attride-Stirling et al. [55] UK	Individual interviews Thematic analysis	11 parents who completed treatment 7 parents who dropped out from treatment	Barriers to continued engagement Parental accounts of why they completed or discontinued treatment within CAMHS	*Reasons for dropping out identified by non*-*completers*: Multiple, personal, parenting and child problems No support network Little understanding of treatment Programme added to stress	*Reliability*: Interview schedule Interviews recorded and transcribed *Validity* Not stated	*Reliability*: Use of transcribed data *Validity*: 3 researchers independently coded. Interrater reliability check on 6 interviews (89 % agreement)	Robust
V Sarno Owens et al. [56] USA	Focus groups Not directly stated (applying the Focus Group Toolkit by Morgan and Kruger [63])	15 parents from a rural, Appalachian area who completed a parenting programme	Barriers to service access Hard to reach groups (rural) Parent’s views of behavioural parent training programme in a rural community	*Barriers*: Programme content & set-up Psychological barriers *Facilitators*: Group support system Therapist qualities & background Programme content & set-up Advertising (testimonials, word of mouth)	*Reliability* Interview schedule developed using the Focus Group Toolkit (Morgan and Kruger [63]) Focus groups recorded and transcribed *Validity*: Moderator fully trained but unaware of the research hypothesis, mock focus group with students conducted before data collection.	*Reliability* Use of transcribed data *Validity*: 1/3 of interviews double-coded, interrater reliability check performed, inconsistencies checked independently.	Robust
VI Cortis et al. [57] Australia	Individual interviews Thematic analysis	120 professionals across 10 sites within child and family services in Australia	Barriers to service access Hard to reach groups Issues of accessibility to children and family services	*Barriers* Psychological barriers (stigma) Situational barriers (transport & location) Difficulties with inter-agency working *Facilitators*: Programme content & set up Good recruitment strategies Provision of resources (food & transport) Positive relationship with therapist	*Reliability* Interview schedule *Validity* Not stated	*Reliability* Not stated *Validity* Not stated	Robust
VII Barrett [58] UK	Semi-structured interviews Thematic Content Analysis	10 frontline managers 10 strategic managers in the Voluntary and Community Sector in rural and urban setting	Overcoming barriers to service access Hard to reach groups (rural) Looks at challenge of delivering services to ‘hard to reach’ families.	*Facilitators*: Multiple access points More information, advice and training in the use of parenting programmes Good organisational structures and staffing Flexible and diverse staff Interagency collaboration	*Reliability* Interview schedule Interviews recorded and transcribed *Validity* Interview questions developed from literature review and based on previous study with hard to reach groups	*Reliability* Use of transcribed data *Validity* Not stated	Robust
VIII Pullmann et al. [60] USA	Individual interviews Grounded Theory	8 caregivers (4 involved with services, 4 not involved or dropped out early) 9 professionals (3 family support providers, 3 community liaisons, 1 programme evaluator, 1 programme marketer, 1 support staff)	Barriers/facilitators to service access Hard to reach groups (rural areas) Barriers to services within a rural system of care site	*Barriers*: Stigma/close knit community Lack of resources (e.g. transportation, money) Lack of knowledge of mental health issues Isolation *Facilitators*: Social support Provision of resources Family/child emotional support Outreach	*Reliability* Interview schedule Interviews recorded and transcribed *Validity* Interview schedule design with various stakeholders	*Reliability* Use of transcribed data *Validity* Coding by two researchers, triangulation, frequent discussions of coding in team	Robust
IX Berlyn et al. [61] Australia	Individual interviews Focus groups Thematic analysis	17 professionals (service managers, project facilitators and workers) 34 fathers	Barriers/facilitators to service access Hard to reach groups (fathers) Strategies to enhance involvement	*Barriers* Mother oriented service culture Lack of knowledge of services Competing work demands Transportation barriers *Facilitators* *1.* Good recruitment strategies Word of mouth Effective marketing and promotion strategies *2.* Good practice in programme delivery Rapport building through sharing experiences Focus on strengths Anti-expert approach Accommodating male learning/communication styles Male friendly spaces, male specific programmes Relaxed and welcoming atmosphere Flexible hours Additional incentives	*Reliability* Interviews recorded and transcribed *Validity* Not stated	*Reliability* Use of transcribed data *Validity* Two researchers coded the data	Robust
X Friars and Mellor [62] Australia	Individual interviews not stated	9 parents of children diagnosed with OCD, CD or ADHD (4–11 years) who dropped out of parent training programmes	Barriers to continued engagement Reasons for dropping out of parenting programmes	*Reasons for dropping out* Child more/less difficult than others in group Programme added to stress levels of parents, practical issues Having several children with problems “Not a group person” Difficulties with strategies Issues with therapist	*Reliability*: Interviews recorded and transcribed by an independent person *Validity*: Not stated	*Reliability*: Use of transcribed data *Validity*: Not stated	Less robust
XI Patterson et al. [29] UK	Individual interviews Grounded Theory	26 parents of children (2–8 years) scoring above the mean on Eyberg, after completion (or dropping out) of IY programme, exclusion if learning difficulties	Barriers to continued engagement Explores reasons for dropping out	*Reasons for dropping out* Aspects of programme delivery Needs not met by programme	*Reliability* Interviewers had access to each other’s recordings to ensure consistency in style and content. Interviews recorded and transcribed *Validity*: Independent interviewers (not involved in IY)	*Reliability*: Use of transcribed data *Validity*: Triangulation	Robust
XII Barrett [59] UK	Individual interviews Analytic approach not stated	24 professionals experienced in delivering parenting	Overcoming barriers to service access Barriers to continued engagement Hard to reach groups	*Recommendations*: Matching parents to programmes Preparing parents Creating a safe space Providing additional support Adopting a collaborative approach Tailoring the programme Interagency work Ensuring facilitators are highly skilled	*Reliability* Interviews recorded and transcribed *Validity* Not stated	*Reliability* Use of transcribed data *Validity* Not stated	Less robust

### Data extraction and synthesis

A thematic synthesis approach was employed [38]. This has three partly overlapping stages: (1) free line-by-line coding of the *Findings* section of primary studies, (2) organisation of these codes into related areas to construct ‘descriptive’ themes and (3) development of ‘analytical’ themes. All included papers were uploaded into *Atlas.ti* (Version 6.2.27). Coding and descriptive thematic development from the *Findings* sections of papers by JK and ES was supervised by SL and discussed at regular team meetings within the research team as a whole. During the process of initial free line-by-line coding every sentence had at least one code applied to it, and most were categorised using several codes. As new papers were coded descriptively, themes were translated from one study to the next and new codes were added to the ‘code bank’ as appropriate. Codes were then reviewed and grouped hierarchically. New codes were created to capture the meaning of clusters of initial codes, forming a tree structure of descriptive themes with several layers. A report of interim findings including the coding framework was produced and circulated within the research team for feedback and validation of the themes. The *Findings* sections for all papers were then re-coded with this coding framework.

## Results

The findings are presented in terms of four emergent core concepts: (1) barriers to service access; (2) barriers to continued engagement; (3) facilitators of service access; and (4) facilitators of continued engagement (see Table 2 for a summary). The views of parents and professionals are presented alongside each other and, where appropriate, attention is drawn to similarities and differences of views held by these two groups for each of the four core concepts. Study-specific references are represented by their corresponding numbers throughout the “Results” section. Issues arising under the major themes and sub-themes constituting each core concept are presented below and more fully in Appendix II, Tables 1–4 (available online).

**Table 2:** Summary of results

	Barriers	Facilitators
Service access	1. Situational barriers • Practical issues (e.g. transport, childcare, inconvenient timing/venue) • Time constraints due to other commitments (e.g. work, having several children) 2. Psychological barriers • Fears/Worries (e.g. confidence, fear of being judged) • Stigma (e.g. shame about needing help, being labelled) • Distrust (e.g. concerns about confidentiality/anonymity) 3. Lack of information/misconception about services (e.g. unawareness of service) 4. Availability of services (e.g. long waiting lists) 5. Poor interagency collaboration (e.g. unorganised referral routes)	1. Effective Advertisement/service promotion • Multi-channel promotion (e.g. leaflets, posters, internet, newsletters) • Effective advertisement content (e.g. clear, easy to understand) • Targeting of hard to reach groups (e.g. wording, images) • Offer of multiple, ‘soft’ entry points (e.g. open events) 2. Direct recruitment • Personalised recruitment (e.g. through good relationship with parent) • Effective, direct channels (e.g. word of mouth between parents) 3. Good interagency collaboration (e.g. multiple referral routes)
Continued engagement	1. Dislike of group activities (e.g. feeling an outsider, shyness) 2. Perception that programme is unhelpful (e.g. programme adding to stress levels) 3. Difficulties following the programme (e.g. lack of support) 4. Change in circumstances (e.g. illness of family member)	1. Programme factors • Programme meets families’ actual needs (e.g. flexible, individually tailored) • Positive group experience (e.g. homogenous groups) • Additional contact (e.g. phone support) 2. Therapist factors • Positive personal qualities of therapist (e.g. non-judgemental, warm) • Therapist skills/background (e.g. continued training)

### Barriers to service access

Twenty-eight issues were identified and these were grouped into five major themes: ‘situational barriers’, ‘psychological barriers’, ‘lack of information/misconception about services’, ‘availability of services’ and ‘poor interagency collaboration’. Overall, parent and professional studies covered these themes to an equal degree; however, there were differences between the parents and professionals views within the sub-themes. These are discussed below.

#### Situational barriers

##### Practical difficulties (transport; childcare; financial difficulties; location; inconvenient timings; unpleasant venue; parking)

Practical issues were reported by all but two [III, XI] studies with the majority reporting difficulties with transport [I, II, VI, VIII–X, XII]. This was particularly problematic for parents living in remote areas [II, VI], for pregnant women and for families with several children [VI]. About half of the parent studies and two professional studies reported difficulties with childcare, financial issues and location as barriers. Financial difficulties, particularly in rural areas [II, VIII], were raised as was attendance associated both with time off work and transportation costs.

##### Time constraints due to other commitments (work; issues associated with having several children)

Time constraints were reported in all parent studies but just three professional studies [III, VI, XII]. The majority highlighted work issues [II–IV, IX–XII] and about half noted families having several children as a barrier [I, II, VIII, X].

#### Psychological barriers

##### Fears/Worries (lack of confidence; shyness; concern about being judged; concern about not having skills)

Half of both parent [I, V, VIII, IX] and professional [I, III, VI, XII] studies reported parental fears and worries–about the unknown, about going to a new programme, and about walking into a new environment. This was related to a lack of confidence [I, XII], shyness [V, VI, IX, X], worrying about being judged [V, VIII] and/or having to share emotions with a wider group of parents—a fear expressed especially by fathers [IX]. Fathers also expressed the worry that they might not have the skills required to follow a programme [III].

##### Stigma (shame about needing help; service use perceived as parental failure; fear of being labelled)

About half of the professional studies [VI–IX] but just two parent studies [II, VIII] identified stigma attached to service use, with the shame about needing or having to ask for help as the most commonly cited issue [II, VI, VIII, IX]. Using services was seen as associated with admitting to being a failure as a parent [V, IX], and with a worry about being labelled [II]. Whilst it was acknowledged that obtaining a diagnosis for the child facilitated getting support, there was a concern that they would be labelled permanently. This was particularly relevant to members of close-knit communities (i.e., rural areas, small towns or religious communities [II, VIII]).

##### Distrust (concern about lack of confidentiality/anonymity; distrust of professionals)

Under this sub-theme, three parent studies [II, V, VIII] but only one professional study [VI] indicated concern about a lack of confidentiality/anonymity in groups, as well as concern about being reported to child protection agencies, especially if they used “corporal punishment” [V, VI] or if parents had been previously involved with the justice system [VI]. These issues were again particularly common amongst members of close-knit communities [II, V, VIII]. Two parent studies [II, V] and one professional study [VI] reported a lack of trust of professionals who were described by parents in one study as “*claimed experts*” [V, p. 188]. Distrust was reported particularly in situations where professionals were from a different cultural or ethnic background to that of the parents [V].

##### Lack of information/misconception about services (unawareness of services; misconceptions about services; belief that there is no need for treatment; advertising insufficient; perception that services are for ‘others’)

These issues were discussed in half of the parent [I–III, IX] and all but two [VI, VII] of the professional studies. Lack of awareness of existing services, the most frequently cited issue across parent studies [I, II, III, IX], was related to insufficient or ineffective advertising and it was suggested that services only reached those proactively seeking help [IX]. Parental misconceptions about the nature/content of services was also mentioned [III, V, IX], such as programmes dictating to families how they should parent, or that available services were intended for “other” parents, the “less fortunate” in the community or “those who cannot cope” [IX, XII]. Not recognising the need for treatment was the most frequently cited issue in professional studies [VIII, IX, XII], related to either “denial” of the problems or, a general lack of knowledge about mental health issues [VIII].

#### Availability of services (limited availability; long waiting time; needs not recognised by professionals; assertiveness of parents, have to be very vocal to get help)

Two parent [I, II] and two professional studies [I, VI] reported that limited availability of programmes resulted in long delays for access or, in rural areas, in out-of-town referrals [II]. The same parent studies also cited professionals’ failure to recognise parents’ need for support (these were parents of children who at the time of interview were formally diagnosed and/or in mental health treatment). According to parents, even when services were available, professionals often associated the child’s behaviour with a normal developmental phase and only through being assertive did parents receive service support. Parents felt that this process was difficult because DBPs are not as conspicuous and easily identifiable as physical illness [II].

#### Poor interagency collaboration (poor/unorganised referral routes; poor communication/sharing of information between agencies; Inappropriate referrals, i.e. mismatch parent programme)

Two parent [I, II] and three professional studies [I, VII, XII] described poor interagency collaboration as a service barrier due to ineffective (e.g. disorganised) referral routes [I, II, XII] or due to poor sharing of information and communication between agencies [I]. In addition, two professional studies argued that referring families inappropriately to programmes could lead to a mismatch between parent and programme, which, they argued, could potentially make parents feel more inadequate and could cause premature dropout from services [VII, XII].

### Barriers to continued engagement

Overall barriers to continued engagement received less coverage in the qualitative literature compared with barriers to access. Eleven issues were identified and these were grouped into four major themes of ‘dislike of group activities’, ‘programme regarded as unhelpful’, difficulties following the programme’ and ‘change in circumstances’. These themes received much more coverage in the parent literature than the professional literature.

#### Dislike of group activities (feelings of being an outsider in the group; difficulties talking in front of group/not a ‘group person’; participation of group members inconsistent)

Four parent studies [III–V, X] and three professional studies [III, IX, XII] reported group issues as reasons for dropping out prematurely. This was reported to be due to feeling like an outsider in the group [III, IX, X, XII], for instance due to cultural differences [II], and/or differences in the severity of the child’s problems [X]. This was particularly important for members of “hard to reach” groups, fathers [III, IX] and families living in rural areas [II, VI].

#### Programme regarded as unhelpful (programme adding to stress levels rather than reducing them; disagreement with strategies; strategies already applied by parent)

Three parent studies [IV, X, XI] but none of the professional studies indicated that PT programmes were regarded as unhelpful due to a belief that the problems were within the child, and so a child-focused intervention would be more effective. Two studies [IV, X] mentioned that the programme was adding to their stress levels rather than reducing them. Other parents disagreed with the programme strategies [IV], or felt that they were already applying them [XI].

#### Difficulty following the programme (no support from other family members; insufficient understanding of content; difficulties with strategies/exercises)

Three parent studies [III, IV, XI] described the difficulties with trying to follow the programme. These included not receiving the necessary support from other family members [III, X] resulting in inconsistent application of strategies, not understanding the programme sufficiently [IV], having difficulties with the weekly exercises or putting the strategies into place [X]. This latter issue also emerged from one professional study [III].

#### Change in circumstances (illness of any family member; move to a different area)

Two parent studies [X, XI] but no professional study reported that a change in circumstances, such as moving away from the area [XI], or missing sessions due to circumstances such as illness of a family member [X] were likely to result in parents dropping out because of a feeling that they have missed too much of the programme to be able to return.

### Facilitators to service access

Twenty-six issues were identified and these were grouped under the three major themes of ‘effective advertisement/service promotion’, ‘direct recruitment’ and ‘good inter-agency collaboration’. Overall, this area received much greater coverage from the professionals compared to parents.

#### Effective advertisement/service promotion

##### Multi-channel promotion (leaflets/posters in locations visited by parents; promotion on the internet; local newspaper/radio stations; post/newsletters; parenting forums)

Four professional studies [III, VI, VII, XII] and one parent [V] study recommended that programmes should be continuously promoted through multiple channels, such as leaflets/posters distributed in locations routinely visited by parents [III, V, VI, XII]; the internet—especially when recruiting fathers [III, XII]; local newspapers or local radio stations [V, XII]; post or newsletters [III, XII]; and parenting forums [XII].

##### Effective advertisement content (clear, easy to understand—regardless of literacy levels; conveyance of tangible benefits of programme and inclusive nature of services)

All but two [VII, VIII] professional studies, but only one parent study [I] recommended that advertisements should convey sufficient information about the nature and type of programmes available in a clear, user-friendly way, accessible to all parents regardless of literacy skills. The professional literature also recommended that service promotion should further explicitly express the tangible benefits of services, in particular if the advertisement is aimed at fathers [III, VI, IX] and it should convey the message that programmes are not only suitable for parents who ‘cannot cope’ but rather emphasise the inclusive nature of services that can benefit everyone [VI, XII].

##### Specifically target hard to reach groups (choice of appropriate advertisement locations; wording/images relevant to specific groups; visual material (e.g. for parents with literacy issues); translation of information for CALD parents; outreach for remote areas through satellite/video)

All but two [II, VIII] professional studies, but none of the parent studies, suggested that ‘hard to reach’ groups should be specifically targeted using tailored advertisement, as they may feel that universal service promotion approaches are not relevant to them [III, VI, IX]. However, it was also reported that this strategy of targeting specific groups might inadvertently exclude other, potentially vulnerable, groups as a consequence [VI]. Approaches targeting specific groups should include appropriate advertisement locations, wordings and images relevant to the specific target group [III, VI, IX], using translation services for parents from CALD backgrounds [VI, VII] and/or using alternative channels, such as visual material [III, VI, XII] to reach parents with literacy problems or parents from CALD backgrounds and outreach for remote areas using satellite/video [VI, XII].

##### Offer multiple, ‘soft’ entry points (fun unrelated events [‘backdoor access’]; open events)

Half of the professional studies [VI, VII, IX, XII] but none of the parent studies recommended that in order to increase accessibility, services should be available through multiple “soft” entry points. Open events such as course taster sessions, coffee mornings/open days to give parents the opportunity to familiarise themselves with the venue and the staff were recommended [VI, XII]. Fun events not directly related to the programme, such as day trips, were suggested to give parents “backdoor access” to services, allowing opportunity to enquire about services in their own terms without feeling stigmatised or blamed [VI, VII, IX].

#### Direct recruitment

##### Personalised recruitment (good relationship with the parent; from similar background as parent; good preparatory work)

All but two [I, VIII] professional studies and three parent studies [II, V, IX] recommended that recruitment should be individually targeted towards specific families. Having a good relationship with the target family was believed to be the key to successful engagement [II, VI, VII, XII], and time and effort should be put into building up relationships prior to the start of the programme, especially when working with vulnerable groups [VI]. Having therapists from a similar background as the parent (e.g. age, ethnicity and/or class) was believed to facilitate this process [III, VII]. Good preparatory work in the form of pre-group sessions was also believed to be useful [VI, XII] to encourage and reassure parents and provide the opportunity to voice any concerns or questions about the programme.

##### Effective, direct channels (other parents/word of mouth; outreach work; emails; phone calls; text message)

Almost half of both the professional [III, VI, IX, XII] and parent [II, III, V, IX] studies indicated that the most effective way of directly recruiting parents was believed to be through other parents who had already completed the course (e.g. “word of mouth” or as parent advocates). Professionals [VI–VIII, XII] further suggested tailored outreach work in the form of home visits for specific families; where, for example, visual materials (such as video clips) can be used with families with literacy issues [XII]. Other recommended channels were emails, phone calls and/or text messages [III].

#### Good interagency collaboration (good, multiple referral routes; updating and training of other agencies about services)

All but two [III, VIII] professional studies but only one parent study [V] suggested good interagency collaboration was needed to improve service accessibility, particularly through multiple, well-organised referral routes [V, VI, IX, XII]. Multiagency work was believed to be particularly important for hard to reach families [VII, IX, XII]. In order for agencies to work together successfully, it was considered important for service providers to inform and continually update other agencies about available programmes [VI, XII].

### Facilitators to continued engagement

Twenty-one issues were identified and these were grouped under two major themes; ‘programme factors’ and ‘therapist factors’. These issues were widely discussed in professional studies but less so across parent studies.

#### Programme factors

##### Programme addresses families’ actual needs (tailoring of flexible programmes specifically towards family; accommodation of different learning/interaction styles; accommodation of special needs; thorough assessment of actual needs; provision of necessary resources)

Half of the parent studies [I–IV, IX] and all but one [VIII] of the professional studies suggested that it was crucial for available programmes to meet families’ actual (rather than perceived) needs and to offer programmes that are flexible enough to be specifically tailored towards each individual family. Factors identified included flexible locations and timings—especially for fathers [III, IX], the accommodation of different learning or interactional styles [I, VI, IX, XII], and the accommodation of special needs by, for instance, involving other agencies if necessary [XII]. In order to individually tailor the programme and to assess whether the programme would benefit the family or not it was believed necessary to thoroughly assess each family’s needs at the beginning of the programme [I, III, VI, VII, XII]. This sub-theme was given the most coverage in the professional literature.

##### Positive group experience (homogenous groups; establishment of ground rules [e.g. confidentiality, safety]; provision of food)

Because sharing experiences and getting support from other group members was regarded as invaluable [XII], it was suggested (both parent and professional literature) that having homogenous groups (e.g. with parents coming from similar backgrounds) was beneficial [V, VI, IX, XII, XII] and establishing ground rules at the beginning of each session was deemed important [V, XII]. Of these rules, confidentiality and a non-judgemental approach were emphasised to help parents share their experiences with the group [XII]. Two studies in the professional literature [VI, IX] also recommended providing food during the group sessions.

##### Additional contact (home visits or one-to-one support; phone support; catch up sessions if any were missed)

Between-session contact for parents was recommended by one parent [II] and three of the professional studies [I, VI, XII]. Recommendations included home visits and one-to-one support [I, II, VI, XII], especially for very complex cases [XII]. Other suggestions were to provide additional phone support [XII], or to offer catch up sessions if any sessions were missed [XII].

#### Therapist factors

##### Positive personal qualities of therapist (ability to build good relationship with parents; importance of personal qualities [non-judgemental/non-patronising; warm/friendly/empathic/caring; flexible/adaptable; collaborative; down to earth/on one level with parents]

All but one [VIII] parent studies and all but two [VII, VIII] of the professional studies mentioned the issues of positive personal qualities of the therapist when discussing factors that promote continued engagement. These included the ability to build good relationships with parents and to facilitate good relationships between group members [I–IV, VI, IX, XII]. Other desirable characteristics were emphasised in all but two [IV, VIII] studies in the parent literature such as the therapist being non-judgemental and non-patronising and/or warm, friendly, empathic and caring and in four professional studies [III, VI, IX, XII]. Other qualities were, being flexible and adaptable [I, II, IV, VI], collaborative rather than authoritarian [V, VI, XI, XII] and on one level with the parents (‘down to earth’) [I, IX].

##### Therapist skills/background (similarities with parents helpful; continued training in wide range of skills; importance of relevant personal experiences; negative connotations with job titles)

The issues under this sub-theme were given more prominence in the professional literature with all but one [VIII] study discussing this theme. Professionals felt that it was important for practitioners to be from a similar background as parents [III, VI, VII, IX, XII] and to have received extensive training in various different skills, to undertake continued professional development, and to receive continued support and supervision [I, III, VI, VII, XII]. Parents, however, felt that professional background and training were irrelevant but rather that the therapist’s personal experience was important, for example of having a disruptive child [I, V].

### Comparison of the parent and professional reports

A synthesis of the findings identified different patterns of responses from parents and practitioners. Overall the parent literature focused more on barriers to both accessing and engaging with services while the professional literature had a more balanced focus across barriers and facilitators. Clear differences emerged between parent and professional perspectives regarding certain themes and these are discussed below.

The parent and practitioner literature covered the core concept of ‘barriers to access’ to an equal degree. However, there were qualitative differences regarding the types of issues raised within a number of themes. The ‘situational barriers’ theme showed that parents placed a greater emphasis on work-related issues and the constraints imposed by having several children to cope with. Such issues received less coverage within the professional literature. Differences also emerged within the ‘distrust’ theme. This was discussed in half of the parent studies but in only one of the professional studies, where the emphasis overall was more on issues related to service provision.

‘Facilitators to service access’ also received a broad coverage in the professional literature but little coverage in the parent literature. However, none of the parent studies mentioned the sub-themes ‘specifically targeting hard-to-reach groups’ and ‘offering multiple ‘soft’ entry points’, and only one study reported on ‘multichannel promotion’ and one study on ‘effective advertisement content’. ‘Barriers to continued engagement’ received more coverage in the parent literature with none of the professionals mentioning the sub-themes ‘programme regarded as unhelpful’, or ‘change in circumstances’, and just one study reporting ‘difficulties following the programme’. Overall ‘barriers to continued engagement’ received much less coverage than ‘barriers to access’ in both the parent and professional literature.

‘Facilitators to continued engagement’ received slightly more coverage in the professional literature compared to the parent literature. The parent literature focused relatively little attention on the sub-theme ‘programme factors’ and more on ‘therapist factors’ whereas the professional studies gave rise to a broad discussion relating to both ‘programme’ and ‘therapist’ factors.

## Discussion

Whilst the efficacy of behavioural PT programmes for the treatment of DBPs in young children has been well established, low take-up and high drop-out rates pose significant threats to their effectiveness within the community setting. The aim of this systematic review was to carry out a thematic synthesis of the qualitative evidence of parent and professional views on accessing and engaging with such programmes and services. Our practical goal was to provide a resource for clinicians and service organisers to promote a more effective implementation of PT programmes.

Multiple barriers and facilitators were identified representing views of both parents and professionals across a range of different nationalities and populations. With regard to accessing services and PT programmes a number of commonly recognised barriers to participation were identified. These included a range of situational factors (e.g. transport and childcare problems, inconvenient timings), several psychological factors (fear, stigma and distrust), unawareness or unavailability of programmes and issues with poor interagency collaboration. Barriers to continued engagement focussed more on group issues, perceiving the programme to be unhelpful, difficulties following the programme and changes in family circumstances.

Our findings were in large part consistent with the *barriers*-*to*-*treatment* model proposed by Kazdin et al. [43, 44]. The model conceptualises barriers to children’s mental health treatment in terms of four main factors: practical obstacles; perceptions that treatment is too demanding; perceptions that treatment is of little relevance to the child’s problems; and poor relationship or alliance with therapist. The first factor, practical obstacles, relates to our sub-theme ‘situational barriers’, the second and third factors relate to our sub-themes ‘difficulty following the programme’ and ‘programme regarded as unhelpful’, and the fourth factor, poor relationship or alliance with therapist, relates to our ‘therapist factor’ sub-theme. Our results also highlighted some additional areas not covered in the model. These included psychological issues (fear, stigma and distrust) and issues specifically relating to group-based programmes. A number of facilitators were also identified as factors that help parents in both accessing and maintaining engagement with services. With regards to accessing treatment, effective advertisement and service promotion (e.g. multi-channel promotion, multiple entry points), direct recruitment (personalised and effective) and good interagency collaboration were identified. A number of programme factors (e.g. programme meeting families’ actual needs) and therapist factors (e.g. personal qualities and professional skills) were suggested in order to help parents maintain engagement with the treatment programme.

When comparing the views of parents and professionals, the parents tended to focus more on the barriers they face regarding accessing and engaging with services, giving less suggestions as to what could help overcome such difficulties. A number of sub-themes, however, were covered mainly within the parent literature, indicating a need for raising greater awareness amongst professionals of such issues. These specifically included ‘time constraints due to other commitments’, ‘distrust’, ‘programme regarded as unhelpful’, ‘difficulties following the programme’ and ‘change in circumstances’. It is important for professionals to gain a better understanding of the parents’ own views in order to better support families, and to offer programmes that are family centred and responsive to the situational and other barriers identified above.

In addition, parent motivation levels may also be linked with the perception of barriers to treatment. This was found in a previous study where increases in parent motivation predicted the perception of fewer barriers to treatment participation and greater treatment attendance [45]. Prochaska and colleagues’ Stages of Change Model [46] describe a spiral pattern of behavioural change comprising of five major stages; precontemplation, contemplation, preparation, action and maintenance with those who are in the early stages being either unaware, in denial or not able to commit to making any changes. As behavioural PT programmes require the parent to make changes to their own behaviour in order to help the child, the parents’ social cognitions and motivational readiness may also impact on both accessing and engaging with services [45, 47]. Parents’ motivational readiness should therefore also be explored with a view to incorporating elements of Motivational Interviewing [48] or other such techniques to help enhance motivation levels where necessary.

## Recommendations and implications for clinical practice

Each family has its own unique characteristics and the current synthesis demonstrates that the widely varying circumstances facing families need to be considered when recruitment and engagement strategies are being developed. There is a clear need for assessment tools that can be used to collect information about specific barriers affecting individual families so interventions can be tailored to the particular needs of the family. In addition it is important to consider parents’ preferences with regards to information dissemination, content and service delivery. Recent use of consumer modelling techniques may prove helpful in this area [49]. Programme developers must be aware that programmes need to be designed to be flexible and accommodate a variety of need, and should be based on what parents want and can realistically manage. PT programmes with particular components might also need to be targeted to groups of parents with particular needs or with children with particular needs. Crucially clinical guidelines, such as the National Institute for Health and Clinical Excellence (NICE) guidelines [3, 50] should address the issue of barriers and facilitators to therapies and not just the efficacy of the treatment itself. It is our hope that the themes identified in the study will help inform the development and delivery of future parenting programmes and be included in treatment guidelines; these include:

1. *Ensuring Awareness and Availability of Services:* Raising the general awareness of programmes and services within the community through good publicity is essential. Tailoring recruitment methods and materials is required in order to attract specific under-represented groups (e.g. fathers, minority groups) who may not be able to identify with general advertisement. Word of mouth is a particularly helpful strategy to attract parents, in particular regarding hard-to-reach groups and therefore involving parents in the recruitment process would therefore be beneficial (e.g. using parent testimonials in recruitment materials, holding coffee mornings or open days, linking with outreach workers within the community).
2. *Creating Individually Tailored Support:* Parents highly value programmes that are flexible and individually tailored. In order to do this the families’ needs should be thoroughly assessed at the outset and any additional support that may be needed provided. This might include linking up support from multiple agencies, providing transport, additional contact between sessions, etc.
3. *Increasing Therapist Skills and Matching them to programmes and families:* Therapists need to be highly skilled, continually trained and knowledgeable across a wide range of areas in order to address the wide variety of individual needs within each family. Adopting a non-judgemental, empathetic and empowering approach is essential with regards to fostering good relationships. It is also recommended, wherever possible, that professionals share some similarities with parents in order to overcome the distrust often initially experienced by parents. Professionals should be aware that distrust can be a significant barrier for parents and therefore developing a trusting relationship is key. This is particularly important when working with hard to reach families.
4. *Making Group*-*based Programmes more acceptable to parents and making available one*-*to*-*one versions of effective programmes:* As group-based parenting programmes are often recommended in the management of DBPs, specifically CD and ADHD [3, 50] it is worth considering a number of issues that have been highlighted specifically relating to such programmes. Parents often highly value the social support they gain from group-based programmes, so ideally programmes should be designed to incorporate aspects that facilitate bonding between group members. However, for some parents group issues are felt to be barriers and reasons for disengaging with treatment. Groups should be kept as homogenous as possible in order to help parents feel like they ‘fit in’ within the group and this is particularly important for underrepresented groups. It’s also crucial that parents feel the group to be a safe, non-judgmental space.

## Limitations and future direction

We acknowledge that the lack of contextual detail may not give justice to the individual studies, but it is largely accepted that the importance of qualitative studies may not be fully recognised if left to accumulate and are not synthesised [51]. Given that the studies selected for synthesis were from different countries, at different time periods, using different populations, and including a range of different family circumstances, the emergence of a number of core themes experienced by *all* the studies allows wider application for the current findings. Several further limitations of the current research must be considered: (1) whilst the synthesis specifically included views of ‘hard to reach’ families, only studies representing fathers, parents from rural areas and from CALD backgrounds were included—views of other ‘hard to reach’ groups are not represented here; (2) the synthesis was limited to interview and focus group studies only and did not include other qualitative methodologies which may help further inform the research; and (3) a large number of the studies were carried out in the UK and this should be taken into account when considering transferability. However, given that the current synthesis remains to our knowledge the first international synthesis of its type, we hope the current findings of this systematic review will help inform the development and delivery of parenting programmes for children with DBPs around the developed world and help open up further lines of academic inquiry relating to the issues raised in the synthesis.

Future research should work to develop instruments that can provide a rapid assessment of the particular requirements of individual families so that interventions can be tailored to their needs, and more qualitative research is needed to help further understand the divergence between parent and service perspectives. In addition, the synthesis of both qualitative and quantitative evidence within this area would be highly valuable.

## Electronic supplementary material

Below is the link to the electronic supplementary material.

Supplementary material 1 (DOCX 19 kb)

Supplementary material 2 (DOCX 53 kb)

## Footnotes

1 For the remainder of the document, the term ‘parent’ refers to both parents and caregivers.
