# Supplementary material for: Barriers to, and facilitators of, parenting programmes for childhood behaviour problems: a qualitative synthesis of studies of parents’ and professionals’ perceptions
Source: Eur Child Adolesc Psychiatry. 2013 Apr 6;22(11):653–70. doi: 10.1007/s00787-013-0401-2 (PMC3826057; doi:10.1007/s00787-013-0401-2)
Supplement: Supplementary file 1 — Supplementary material 1 (DOCX 19 kb) [file 787_2013_401_MOESM1_ESM.docx]

**Appendix I**

**MEDLINE SEARCH STRATEGY (search conducted August, 2010)**

1. Child$ or infant$ or preschool$ or pre-school$ or pre-school$ or Kindergarten or early year$ or pre-five or pre five

2. (Attention adj3 Hyperactiv$) or (attention adj5 deficit) or Adhd or adhkd or addh or adhs or Hyperactive$ or hyperkine$ or child$ psychopathol$ or pervasive development$ or PDD or Child development disorder$ or Autis$ or Asperger$ or ASD or ASC

3. (opposition$ or conduct or behavio?r$ or antisocial or anti-social or emotion$ or disruptive or violen$ or anger or externali?ing or aggres$ or attachment or separation or psych$ or emotional or mental or anxiety or mood or personality or developmental or eating or language or learning or neurotic or speech or communication or sleep$ or internali?ing) adj3 (defiant$ or disorder$ or acilitate$ or problem$ or difficult$ or symptom$ or behavio?r$)

4. Mental$ adj4 (health or disease$ or deficien$ or retard$)

5. OR 2-4

6. (parent$ or famil$ or child$ or mother$ or father$) adj3 (program$ or intervention$ or training or education or promotion or treatment or therap$ or service$ or support$ or counsel$ or contingency management)

7. parent$ or famil$ or child$ or mother$ or father$

8. (early or prevent$ or primary or tailor$) adj3 (intervention$ or program$ or training or therapy or service or support or counsel$ or promotion or treatment or education$ or prevention$ or care or strategy)

9. (education$ or train$ or treat$) adj2 (program$ or intervention or outreach or strategy or service)

10. (management or skill$) adj3 training

11. AND 7 (OR 8 -10)

12. 6 OR 11

13. ((parent$ or famil$ or child$ or mother$ or father$) adj3 (program$ or intervention$ or training or education or promotion or treatment or therap$ or service$ or support$ or counsel$ or contingency management)) adj6 (access$ or engag$ or acilitate$ or help$ or assist$ or admission or admit$ or Utili?e or Barrier$ or obstruct$ or hurdle$ or difficult$ or imped$ or hindrance or Refus$ or decline$ or reject$ or resist$ or stigma$ or perception$ or acilita$ or belie$ or cognition$ or acilitat$ or acilita$)

14. ((parent$ or famil$ or child$ or mother$ or father$) adj3 (program$ or intervention$ or training or education or promotion or treatment or therap$ or service$ or support$ or counsel$ or contingency management)) adj6 (retention or maintain or continu$ or Attend$ or Adher$ or Complian$ or Engag$ or Participat$ or Cooperat$ or co-operarat$ or co operat$ or Involv$ or Continu$ or Collaborat$ or therapeutic alliance or Complet$ or Concord$ or persist$ or conform$ or Continu$ or non-attend$ or non-attend$ or nonattend$ or non-accept$ or non-accept$ or nonaccept$ or non adher$ or non-adher$ or nonadher$ or noncomplian$ or non complian$ or non-complian$ or non participat$ or non-participat$ or acilitateant$ or uncooperat$ non acilit$ or non-involv$ or noninvolv$ or non complet$ or non-complet$ or noncomplet$ or non-concord$ or non-concord$ or nonconcord$ or non persisten$ or non-persisten$ or nonpersisten$ or non-conform$ or non-conform$ or nonconform or Disengag$ or Discontinu$ or abstention or abstain$ or stop$ or abandon$ or terminat$ or Attrition or dropout$ or drop out$)

15. ((parent$ or famil$ or child$ or mother$ or father$) adj3 (program$ or intervention$ or training or education or promotion or treatment or therap$ or service$ or support$ or counsel$ or contingency management)) adj6 (**t**herapeutic change or effect$ or effic$ or help$ or success$ or acilit$ or acilit$ or valu$ or acilitate$ or target$ or reduce$ or chang$ or moderat$ or outcome$ or predict$ or influence$ or respon$)

16. OR 13-15

17. 1 AND 5 AND 12 AND 16 (preschool mental health treatments)

18. Poverty or Poor or Disadvant$ or low$ income or Socio economic or socio-economic or socioeconomic or isolate$ or Remote or Cut off or Inaccessible or Marginali$ or Lonely or slip$ through the net or Travel?er$ or Asylum seek$ or Homeles$ or Refugee$ or Rural or underserve$ or at risk or high risk or discriminat$ or exclu$

19. (Soci$ or economic$ or socioeconomic$) adj4 (disadvant$ or exclu$ or problem$)

20. (Indigenous or ethnic or minorit$ or vulnerable or continental or underrepresented or underrepresented or Rac$) adj4 (group$ or population$ or minorit$ or Culture$)

21. (parent$ or famil$ or mother$ or father$) adj3 (stress or competenc$ or dysfunction)

22. (hard or difficult) adj3 (reach or engage or treat or circumstance$ or chang$ OR implement$)

23. (Lone or single) adj3 (parent$ or mother$ or father$)

24. Hous$ adj3 (problem$ or difficult$)

25. OR 18-24

26. 17 AND 25

27. (parent$ or maternal or paternal or mother$ or father$) adj5 (psych$ or mental$ or emotion$ or anxiety or depress$ or substance or alcohol$ or self-esteem or self-esteem or coping or skill$ or disab$)

28. (parent$ or maternal or paternal or mother$ or father$ or adult) adj5 ((Attention adj3 Hyperactiv$) or (attention adj5 deficit) or Adhd or adhkd or addh or adhs or Hyperactive$ or hyperkine$)

29. (parent$ or maternal or paternal or mother$ or father$ or adult) adj5 (learning diff$ or learning problem$ or learning disab$ or retard$ or intellect$ or handicap$)

30. Mother-child relationship/

31. OR 27-30

32. 17 AND 31

33. Complicated$ or complex$ or comorbid$ or co-morbid$ or co morbid or co-occur$ co-occur$

34. 17 AND 33
