# Supplementary material for: Barriers to, and facilitators of, parenting programmes for childhood behaviour problems: a qualitative synthesis of studies of parents’ and professionals’ perceptions
Source: Eur Child Adolesc Psychiatry. 2013 Apr 6;22(11):653–70. doi: 10.1007/s00787-013-0401-2 (PMC3826057; doi:10.1007/s00787-013-0401-2)
Supplement: Supplementary file 2 — Supplementary material 2 (DOCX 53 kb) [file 787_2013_401_MOESM2_ESM.docx]

**Appendix II**

Table 1: Barriers to Service Access

|  | **Parents** | | | | | | | | **Pa / Pr*** | **Professionals** | | | | | |
| --- | --- | --- | --- | --- | --- | --- | --- | --- | --- | --- | --- | --- | --- | --- | --- |
| **Study ID** | 1 | 2 | 4 | 5 | 8 | 9 | 10 | 11 | 3 | 1 | 6 | 7 | 8 | 9 | 12 |
| **Situational Barriers** | **X** | **X** | **X** | **X** | **X** | **X** | **X** | **X** | **X** | **X** | **X** | **X** | **X** | **X** | **X** |
| ***Practical issues*** | X | X | X | X | X | X | X | - | - | X | X | X | X | X | X |
| Transport issues | X | X | - |  | X | X | X | - | - | X | X | - | X | - | X |
| No childcare | X | X | - | X | - | - | X | - | - | - | - | - | - | X | X |
| Financial difficulties | - | X | - | - | X | - | X | - | - | - | - | X | X | X | - |
| Location issues (inconvenient or unsafe) | - | X | X | X | - | - | - | - | - | - | X | - | - | - | X |
| Inconvenient timings | - | - | X | - | - | - | X | - | X | - | - | - | - | - | - |
| Unpleasant venue | X | - | - | - | - | - | - | - | - | - | - | - | - | X | X |
| No parking | - | - | - | - | - | - | X | - | - | - | - | - | - | - | - |
| ***Time constraints due to other commitments*** | X | X | X | X | X | X | X | X | X | - | X | - | - | - | X |
| Work | - | X | X | - | - | X | X | X | X | - | - | - | - | - | X |
| Having several children | X | X | - | - | X | - | X | - | - | - | X | - | - | - | - |
| **Poor interagency collaboration** | **X** | **X** | **-** | **-** | **-** | **-** | **-** | **-** | **-** | **X** | **-** | **X** | **-** | **-** | **X** |
| Poor/unorganised referral routes | X | X | - | - | - | - | - | - | - | X | - | - | - | - | X |
| Poor communication / sharing of info between agencies | X | - | - | - | - | - | - | - | - | X | - | - | - | - | - |
| Inappropriate referrals (mismatch parent – programme) | - | - | - | - | - | - | - | - | - | - | - | X | - | - | X |

Barriers to service access continued

|  | **Parents** | | | | | | | | **Pa / Pr*** | **Professionals** | | | | | |
| --- | --- | --- | --- | --- | --- | --- | --- | --- | --- | --- | --- | --- | --- | --- | --- |
| **Reference ID** | 1 | 2 | 4 | 5 | 8 | 9 | 10 | 11 | 3 | 1 | 6 | 7 | 8 | 9 | 12 |
| **Lack of information / misconceptions about services** | **X** | **X** | **-** | **X** | **-** | **X** | **-** | **-** | **X** | **X** | **-** | **-** | **X** | **X** | **X** |
| Unawareness of services | X | X | - | - | - | X | - | - | X | X | - | - | - | - | - |
| Misconceptions about services | - | - | - | X | - | X | - | - | X | - | - | - | X | - | - |
| Belief that there’s no need for treatment | - | - | - | X | - | - | - | - | - | - | - | - | X | X | X |
| Insufficient advertising | X | - | - | - | - | - | - | - | - | X | - | - | - | - | - |
| Services are for “others” | - | - | - | - | - | - | - | - | X | - | - | - | - | X | - |
| **Psychological barriers** | **X** | **X** | **-** | **X** | **X** | **X** | **-** | **-** | **X** | **X** | **X** | **X** | **X** | **X** | **X** |
| ***Fear/worries*** | X | - | - | X | X | X | - | - | X | X | X | - | - | - | X |
| Lack of confidence | X | - | - | - | - | - | - | - | - | X | - | - | - | - | X |
| Shyness / don’t want to share with group | - | - | - | X | - | X | X | - | - | - | X | - | - | - | - |
| Worry about being judged | - | - | - | X | X | - | - | - | - | - | - | - | - | - | - |
| Worry about not having the skills | - | - | - | - | - | - | - | - | X | - | - | - | - | - | - |
| ***Stigma*** | - | X | - | - | X | - | - | - | - | - | X | X | X | X | - |
| Shame about needing help | - | X | - | - | X | - | - | - | - | - | X | - | X | X | - |
| Using services perceived as admitting to having failed as parent | - | - | - | X | - | - | - | - | - | - | - | - | - | X | - |
| Fear of being labelled | - | X | - | - | - | - | - | - | - | - | - | - | - | - | - |
| ***Distrust*** | - | X | - | X | X | X | - | - | - | - | X | - | - | - | - |
| Worry about lack of confidentiality/anonymity | - | X | - | X | X | - | - | - | - | - | X | - | - | - | - |
| Distrust of professionals | - | X | - | X | - | - | - | - | - | - | X | - | - | - | - |
| **Availability of services** | **X** | **X** | **-** | **-** | **-** | **-** | **-** | **-** | **-** | **X** | **X** | **-** | **-** | **-** | **-** |
| Limited availability / long waiting time | X | X | - | - | - | - | - | - | - | X | X | - | - | - | - |
| Needs not recognised by professionals / parents have to be very vocal to get help | X | X | - | - | - | - | - | - | - | - | - | - | - | - | - |

Table 2: Barriers to continued engagement

|  | **Parents** | | | | | | | | **Pa / Pr*** | **Professionals** | | | | | |
| --- | --- | --- | --- | --- | --- | --- | --- | --- | --- | --- | --- | --- | --- | --- | --- |
| **Reference ID** | 1 | 2 | 4 | 5 | 8 | 9 | 10 | 11 | 3 | 1 | 6 | 7 | 8 | 9 | 12 |
| **Group issues** | - | - | X | X | - | - | X | - | X | - | - | - | - | X | X |
| Feeling like an outsider in the group | - | - | - | - | - | - | X | - | X | - | - | - | - | X | X |
| Difficulties talking in front of group / not a ‘group person’ | - | - | - | - | - | - | X | - | - | - | - | - | - | - | - |
| Inconsistent participation of group members | - | - | - | X | - | - | - | - | - | - | - | - | - | - | - |
| **Programme regarded as unhelpful** | - | - | X | - | - | - | X | X | - | - | - | - | - | - | - |
| Programme adding to stress levels rather than reducing them | - | - | X | - | - | - | X | - | - | - | - | - | - | - | - |
| Disagreement with strategies | - | - | X | - | - | - | - | - | - | - | - | - | - | - | - |
| Already applying strategies | - | - | - | - | - | - | - | X | - | - | - | - | - | - | - |
| **Difficulty following the programme** | - | - | X | - | - | - | X | - | X | - | - | - | - | - |  |
| No support from other family members | - | - | - | - | - | - | X | - | X | - | - | - | - | - | - |
| Insufficient understanding of content | - | - | X | - | - | - | - | - | - | - | - | - | - | - | - |
| Difficulties with strategies/exercises | - | - | - | - | - | - | X | - | - | - | - | - | - | - | - |
|  |  |  |  |  |  |  |  |  |  |  |  |  |  |  |  |
| **Change in circumstances** | - | - | - | - | - | - | X | X | - | - | - | - | - | - | - |
| Illness of any family member | - | - | - | - | - | - | X | - | - | - | - | - | - | - | - |
| Moving away from the area | - | - | - | - | - | - | - | X | - | - | - | - | - | - | - |

Table 3: Facilitators to service access

|  | **Parents** | | | | | | | | **Pa/Pr*** | **Professionals** | | | | | |
| --- | --- | --- | --- | --- | --- | --- | --- | --- | --- | --- | --- | --- | --- | --- | --- |
| **Reference ID** | 1 | 2 | 4 | 5 | 8 | 9 | 10 | 11 | 3 | 1 | 6 | 7 | 8 | 9 | 12 |
| **Effective Advertisement / Service Promotion** | **X** | **-** | **-** | **X** | **-** | **-** | **-** | **-** | **X** | **X** | **X** | **X** | **-** | **X** | **X** |
| ***Multichannel promotion*** | - | - | - | X | - | - | - | - | X | - | X | X | - | - | X |
| Leaflets/posters in locations visited by parents | - | - | - | X | - | - | - | - | X | - | X | - | - | - | X |
| Promotion on the Internet | - | - | - | - | - | - | - | - | X | - | - | - | - | - | X |
| Local newspapers / radio stations | - | - | - | X | - | - | - | - | - | - | - | - | - | - | X |
| Post/newsletters | - | - | - | - | - | - | - | - | X | - | - | - | - | - | X |
| Parenting forums | - | - | - | - | - | - | - | - | - | - | - | - | - | - | X |
| ***Effective advertisement content*** | X | - | - | - | - | - | - | - | X | X | X | - | - | X | X |
| Clear, easy to understand, regardless of literacy levels | X | - | - | - | - | - | - | - | X | X | X | - | - | - | X |
| Convey tangible benefits of programme | - | - | - | - | - | - | - | - | X | - | X | - | - | X | - |
| Convey inclusive nature of services that benefit everyone | - | - | - | - | - | - | - | - | - | - | X | - | - | - | X |
| ***Specifically target hard to reach groups*** | - | - | - | - | - | - | - | - | X | - | X | X | - | X | X |
| Choice of appropriate advertisement locations | - | - | - | - | - | - | - | - | X | - | X | - | - | X | - |
| Wording/images relevant to specific groups | - | - | - | - | - | - | - | - | X | - | X | - | - | X | - |
| Visual material (e.g. for parents with literacy issues) | - | - | - | - | - | - | - | - | X | - | X | - | - | - | X |
| Translating information for CALD parents | - | - | - | - | - | - | - | - | - | - | X | X | - | - | - |
| Outreach for remote areas through satellite/video | - | - | - | - | - | - | - | - | - | - | X | - | - | - | X |
| ***Offer multiple, “soft” entry points*** | - | - | - | - | - | - | - | - | - | - | X | X | - | X | X |
| Fun, unrelated events (‘backdoor access’) | - | - | - | - | - | - | - | - | - | - | X | X | - | X | - |
| Open events | - | - | - | - | - | - | - | - | - | - | X | - | - | - | X |
| **Direct recruitment** | - | **X** | **-** | **X** | **-** | **X** | **-** | **-** | **X** | **X** | **X** | **X** | **X** | **X** | **X** |
| ***Personalised recruitment*** | - | X | - | X | - | X | - | - | X | - | X | X | - | X | X |
| Good relationship with the parent | - | X | - | - | - | - | - | - | - | - | X | X | - | - | X |
| From similar background as parent | - | - | - | - | - | - | - | - | X | - | - | X | - | - | - |
| Good preparatory work | - | - | - | - | - | - | - | - | - | - | X | - | - | - | X |
| ***Effective, direct channels*** | - | **X** | **-** | **X** | **-** | **X** | **-** | **-** | **X** | **-** | **X** | **X** | **X** | **X** | **X** |
| Other parents / Word of mouth | - | X | - | X | - | X | - | - | X | - | X | - | - | X | X |
| Outreach work | - | - | - | - | - | - | - | - | - | - | X | X | X | - | X |
| Emails | - | - | - | - | - | - | - | - | X | X | - | - | - | - | - |
| Phone calls | - | - | - | - | - | - | - | - | X | - | - | - | - | - | - |
| Text messages | - | - | - | - | - | - | - | - | X | - | - | - | - | - | - |
| **Good interagency collaboration** | - | - | - | **X** | **-** | **-** | **-** | **-** | **-** | **X** | **X** | **X** | **-** | **X** | **X** |
| Good, multiple referral routes | - | - | - | X | - | - | - | - | - | - | X | - | - | X | X |
| Especially important with complex needs and hard to reach parents | - | - | - | - | - | - | - | - | - | - | - | X | - | X | X |
| Continually update and train other agencies about services | - | - | - | - | - | - | - | - | - | - | X | - | - | - | X |

Table 4: Facilitators to continued engagement

|  | **Parents** | | | | | | | | **Pa/Pr*** | **Professionals** | | | | | |
| --- | --- | --- | --- | --- | --- | --- | --- | --- | --- | --- | --- | --- | --- | --- | --- |
| **Reference ID** | 1 | 2 | 4 | 5 | 8 | 9 | 10 | 11 | 3 | 1 | 6 | 7 | 8 | 9 | 12 |
| **Programme factors** | **X** | **X** | **X** | **X** | **X** | **X** | **-** | **X** | **X** | **X** | **X** | **X** | **X** | **X** | **X** |
| ***Programme meets families’ actual needs*** | X | X | X | - | - | X | - | - | X | X | X | X | - | X | X |
| Flexible programmes specifically tailored towards family | - | X | X | - | - | X | - | - | X | X | X | X | - | X | X |
| Accommodate different learning/interaction styles | - | - | - | - | - | - | - | - | - | X | X | - | - | X | X |
| Accommodate special needs | X | - | - | - | - | - | - | - | X | X | X | X | - | - | X |
| Thorough assessment of actual needs | - | - | - | - | - | - | - | - | X | X | X | X | - | - | X |
| Provide necessary resources | - | - | - | - | X | - | - | - | - | - | - | - | X | X | X |
| ***Ensure positive group experience*** | - | - | - | X | - | X | - | X | - | - | X | - | - | X | X |
| Homogenous groups | - | - | - | X | - | X | - | X | - | - | X | - | - | X | X |
| Establishing ground rules (e.g. confidentiality, safety) | - | - | - | X | - | - | - | - | - | - | - | - | - | - | X |
| Provide food | - | - | - | - | - | - | - | - | - | - | X | - | - | X | - |
| ***Additional contact*** | - | X | - | - | - | - | - | - | - | X | X | - | - | - | X |
| Home visits or one to one support | - | X | - | - | - | - | - | - | - | X | X | - | - | - | X |
| Phone support | - | - | - | - | - | - | - | - | - | - | - | - | - | - | X |
| Catch up sessions if any were missed | - | - | - | - | - | - | - | - | - | - | - | - | - | - | X |
| **Therapist factors** | **X** | **X** | **X** | **X** | **-** | **X** | **X** | **X** | **X** | **X** | **X** | **X** | **-** | **X** | **X** |
| ***Positive personal qualities of therapist*** | X | X | X | X | - | X | X | X | X | X | X | - | - | X | X |
| Able to build good relationship with parents | - | X | X | - | - | X | - | - | X | X | X | - | - | X | X |
| Non-judgemental / non-patronising | - | X | - | X | **-** | X | **-** | X | - | - | X | - | **-** | - | X |
| Warm / friendly / empathetic /caring | X | X | - | X | - | X | X | X | X | - | - | - | - | X | X |
| Flexible / adaptable | - | X | X | - | - | - | - | - | - | X | X | - | - | - | - |
| Collaborative | - | - | - | X | - | - | - | X | - | - | X | - | - | - | X |
| Down to earth/on one level with parents | X | - | - | - | - | X | - | - | - | - | - | - | - | X | - |
| ***Therapist skills / background*** | X | - | - | X | - | - | - | - | X | X | X | X | - | X | X |
| Helpful if similarities with parents | - | - | - | - | - | - | - | - | X | - | X | X | - | X | X |
| Continued training in wide range of skills | - | - | - | - | - | - | - | - | X | X | X | X | - | - | X |
| Relevant personal experiences important | X | - | - | X | - | - | - | - | - | - | X | - | - | X | X |
| Negative connotations with job titles (e.g. social worker) | X | - | - | - | - | - | - | - | - | - | - | - | - | - | - |

*Parent and professional data was not separated in these studies
